# gp96 Expression in Gliomas and Its Association with Tumor Malignancy and *T* Cell Infiltrating Level

**DOI:** 10.1155/2022/9575867

**Published:** 2022-06-26

**Authors:** Chunzhao Li, Yi Wang, Lang Long, Peng Zhang, Yang Zhang, Nan Ji

**Affiliations:** ^1^Department of Neurosurgery, Beijing Tiantan Hospital, Capital Medical University, Beijing 100070, China; ^2^China National Clinical Research Center for Neurological Diseases, Beijing, China; ^3^China National Clinical Research Center for Neurological Diseases, Beijing Tiantan Hospital, Capital Medical University, Beijing, China; ^4^Beijing Advanced Innovation Center for Big Data-Based Precision Medicine, Beihang University, Beijing, China

## Abstract

Heat shock protein glycoprotein 96 kDa (gp96) implicates in glioma invasiveness and engages antitumor immune response, representing a potential target for glioma treatment. However, its expression in different types of gliomas, its association with glioma-infiltrating *T* cells (GITs), and their clinical significance remain unknown. Herein, we utilized multiplex immunofluorescence staining (MIS) to detect gp96 expression and GIT levels on a tissue microarray (TMA), that comprises 234 glioma cases. We then validated the TMA results and explored possible mechanisms by investigating the RNA-seq data from The Cancer Genome Atlas (TCGA) and the Chinese Glioma Genome Atlas (CGGA). We observed that gp96 was ubiquitously expressed in all types of gliomas whereas overexpressed in grade IV gliomas. Also, high gp96 expression predicted unfavorable outcomes independent of the malignancy grade. Meanwhile, gp96 expression positively correlated CD8^+^, CD4^+^, and PD-1^+^ cell densities, and especially associated with increased infiltration of CD4^+^ PD-1^+^ GITs. Clinically, the gp96-immune cell score (GI score), by summing the values measuring gp96 expression and immune cell densities, is capable of stratifying patients into four outcome-distinct groups (hazard ratio, 1.945; 95% CI, 1.521–2.486; *P* < 0.0001). Mechanistically, the interferon-*γ*/*α* response pathways were revealed to engage in the association between gp96 and GITs. Taken together, gp96 was ubiquitously expressed in gliomas, overexpressed in grade IV gliomas, and increased with GIT infiltrative levels. The GI score, that integrates levels of gp96 expression and GIT infiltration, is a potential prognostic classification system for gliomas.

## 1. Introduction

Malignant glioma is an invasive tumor that accounts for 80% of all primary malignant central nervous system (CNS) tumors [1]. According to the WHO malignancy grade system, malignant glioma is categorized into grade II, III, and IV gliomas; a higher grade usually indicates a higher degree of malignancy and a worse clinical outcome [2]. The standard-of-care treatment for malignant glioma is surgery followed by radiotherapy and/or chemotherapy. However, the treatment outcomes are quite dismal, especially for grade IV gliomas, that include IDH-mutant grade IV gliomas and IDH-wildtype glioblastomas (GBMs). The median overall survival (OS) is limited within 15 months in these patients with grade IV gliomas [3]. Therefore, new therapeutic modalities are urgently needed for this deadly cancer.

Heat shock protein glycoprotein 96 kDa (gp96) is a stress-induced molecular chaperone that belongs to the heat shock protein 90 (HSP90) family. gp96 is usually located in the endoplasmic reticulum (ER), where it physiologically assists in the folding and assembly of membrane or secretory proteins and maintains ER homeostasis. gp96 overexpression has been implicated in the pathogenesis of a variety of malignancies [4–7], including gliomas [8]. It promotes glioma aggressiveness via the Wnt/*β*-catenin signaling pathway, making it a potential molecular target for glioma treatment [8]. Therefore, gp96-selective inhibitors and monoclonal antibodies are under development and have presented promising preclinical outcomes [9–11]. However, there is still a lack of comprehensive knowledge on gp96 expression in different types of gliomas. A better understanding would assist in the selection of appropriate patients for this novel gp96-targeting treatment in future clinical studies.

gp96 is also a key immune molecule that engages in cancer immunity by facilitating antigen presentation [11], assisting in the secretion of proinflammatory cytokines [12] and chaperoning key immune receptors [11, 13]. Therefore, several studies have reported a close correlation of gp96 overexpression with *T* cell accumulation within tumors, such as cholangiocellular carcinoma [14] and lung adenocarcinoma [7]. However, to our knowledge, no such study has been reported regarding gliomas. Moreover, the exact mechanisms and clinical value of this correlation have yet to be explored.

Herein, we utilized multiplex immunofluorescence staining (MIS) and analyzed the RNA-seq data from The Cancer Genome Atlas (TCGA) and Chinese Glioma Genome Atlas (CGGA) to explore the clinicopathological, genetic, and immune features of gp96-expressing gliomas in a large cohort of patients. We then focused on the correlation between gp96 expression and glioma-infiltrating *T* cells (GITs) and investigated the influence of the correlation on the outcomes of glioma patients, as well as explored the possible underlying mechanisms.

## 2. Methods

### 2.1. Patients

This study was supported by the Neurosurgical Clinical Information and Biobanking Project of Beijing Tiantan Hospital (Brain Tumor Section) and was approved by the Ethics Committee of Beijing Tiantan Hospital (KY2014-021-02). A total of 267 pathologically confirmed glioma tissue specimens were obtained from the biobank and were used to construct a tissue microarray (TMA; one tumor core from each specimen). Among these specimens, 234 were evaluable and were included in this study. All specimens were collected from patients who underwent their first microsurgical resection at Beijing Tiantan Hospital. The corresponding clinical information and histological and molecular diagnostic information were extracted from the clinical information system. We also utilized [15] cases of peritumor brain tissues, that were pathologically confirmed as normal brain, to investigate gp96 expression and immune cell infiltration in normal brain tissues. Written informed consent was obtained from all participants.

### 2.2. TCGA and CGGA Analyses

RNA sequencing (RNA-seq) data and related clinical and genetic information were downloaded from the TCGA (www.cgga.org.cn/) on June 9, 2020, and the CGGA (https://www.cgga.org.cn) on May 6, 2020. The analyzed TCGA datasets included 692 glioma samples, whereas the CGGA datasets included 325 samples. The R package DESeq2 was utilized to perform differential expression analysis. Gene set enrichment analysis (GSEA) was then performed on these differentially expressed genes (DEGs) using the hallmark gene sets from the Molecular Signatures Database (MSigDB, v7.2). Genes with fold-changes > 2 and adjusted *p* values < 0.01 between the two groups were defined as DEGs. A total of 24 DEGs that were shared by the IFN-*α* and IFN-*γ* response pathways were selected to build an interferon pathway activity (IPA) score to represent the activities of the IFN-*α* and IFN-*γ* response pathways in each specimen. The IPA score was defined as the arithmetic mean of the log_2_ (transcripts per million (TPM) + 0.1) of 24 shared genes. Each analysis was performed in R 3.6.3.

### 2.3. MIS Assay

We used OPAL™ 7-color Manual IHC kits (NEL811001 KT, Akoya Bioscience, Marlborough, MA, USA) to conduct MIS on the TMA as well as on the FFPE tissues from HSPPC-96-vaccinated patients. After being routinely deparaffinized and rehydrated, the 5 *µ*m slides were subjected to a procedure that was specific for each antigen that was optimized in our preliminary experiments. The detailed experimental procedures and conditions are summarized in Supplemental Table 1. In brief, first, the slides were subjected to microwave-assisted antigen retrieval for 20 minutes at 95°C, and then were blocked in a blocking solution for 10 minutes at room temperature. Second, the slides were incubated in primary antibody solution diluted by the blocking solution according to a specific condition (Supplemental Table 1). Third, after being washed in Tris-buffered saline Tween-20 (TBST, Solarbio, Beijing, China) solution for 10 minutes, the slides were incubated in Polymer HRP Ms + Rb working solution and then in OPAL™ Fluorophore Solution at a dilution of 1 : 100, each for 10 minutes at room temperature. Fourth, the above procedure was repeated until all the antigens were stained. Finally, the slides were routinely stained with DAPI (C0060, Solarbio, Beijing, China) and mounted with mounting medium (VECTASHIELD, Vector, CA, USA).

### 2.4. Multispectral Imaging (MSI) Analysis

Images were obtained by MSI on a Vectra system (Vectra Polaris 1.0.7, Akoya Bioscience, Marlborough, MA, USA) and analyzed by using the Inform software (2.4.2, Akoya Bioscience, Marlborough, MA, USA). The expression of gp96 and GFAP was semiquantified according to the staining extent, which was defined as the number of nuclei of the positively stained cells divided by the number of all nuclei in the section. The density of GITs (CD4, CD8, and PD-1 immune cells) was semiquantified as counts per mm^2^ microscopic field. The staining extent as well as the counts was automatically calculated by using the Inform software and then confirmed under manual inspection by an experienced pathologist. Spatial distribution analysis was conducted using the HALO image analysis software (v3.1, High-plex FL 3.1.0.0, Indica labs, Albuquerque, USA) with assistance from Nanjing Freethinking Biotechnology Co. Ltd. (Nanjing, China).

### 2.5. Statistical Analysis

Unless otherwise specified, all statistical analyses were performed using the SPSS software (version 24.0, SPSS Inc, Chicago, IL). Two group comparisons were performed with the Mann–Whitney *U* test. Multiple comparisons were assessed with a two-sided Kruskal–Wallis *H* test with *p* values adjusted by Bonferroni correction. Spearman correlation analysis was utilized to examine the associations. The method of maximally selected rank statistics was used to set cut-off values for the high and low group of gp96 expression and immune cell densities. Kaplan–Meier analysis was used to estimate OS, and the log-rank test was applied to estimate between-group OS differences. Multivariate Cox regression analysis was utilized to select independent prognostic factors. A 2-tailed *p* value < 0.05 was considered significant. GraphPad Prism 6 (GraphPad Software, Inc, San Diego, CA) and the *R*^2^ package were used to plot the figures.

## 3. Results

### 3.1. gp96 Was Ubiquitously Expressed in Malignant Gliomas and Overexpressed in Grade IV Gliomas

**Figure 1:**
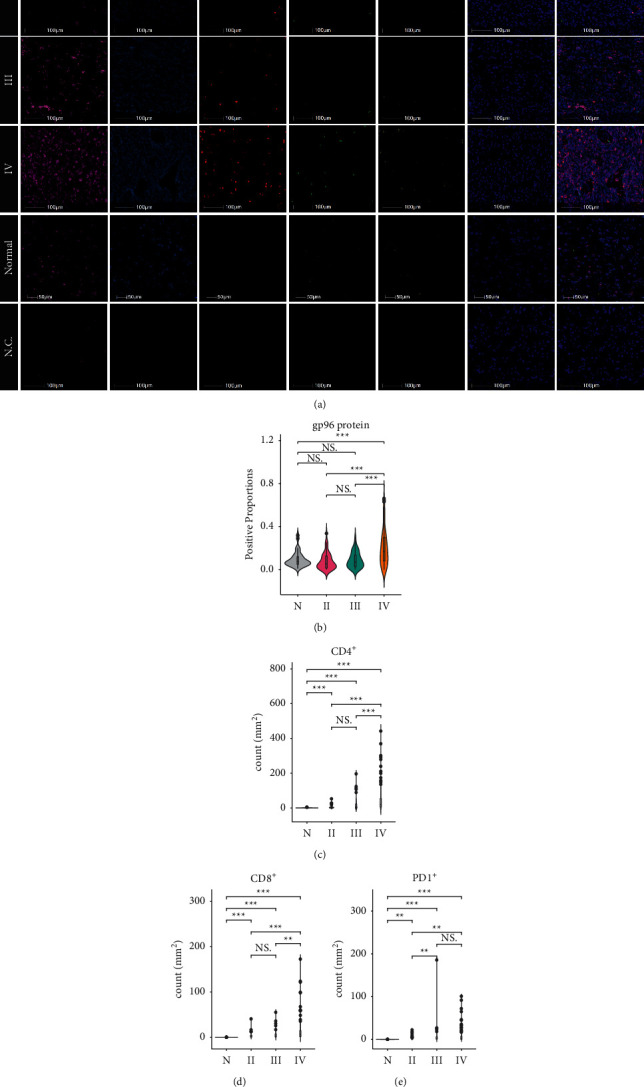
Increased gp96 expression and immune infiltration in WHO grade IV gliomas. (a) Representative staining of gp96, GFAP, CD4, CD8, and PD-1 in grade II to IV gliomas and normal brain tissue (normal). N.C: negative control. (b–e) Comparisons of the extent of gp96 staining and densities of CD4, CD8, and PD-1 infiltrative immune cells among grade II, III, IV gliomas, and normal brain tissues (normal). Kruskal–Wallis test, Bonferroni post hoc method, ^*∗*^*p* < 0.05, ^*∗∗*^*p* < 0.01, ^*∗∗∗*^*p* < 0.001. For better demonstration of the comparison, some specimens with extremely high values (3 in CD4, 3 in CD8, and 5 in PD-1) were discarded in the graphs but were included in the statistical analysis.

There is still a lack of a comprehensive understanding of gp96 expression in a variety of gliomas. Thus, we utilized MIS to detect and semiquantify gp96 expression in samples on the TMA (Figure 1(a)), which consisted of 234 glioma samples: 101 grade II-III and 133 grade IV (GBM) glioma samples (Supplemental Table 2). Among these cases, 90.6% (212/234) had complete follow-up data.

Consistent with its ubiquitous presence in the ER lumen of nucleated cells, cytoplasmic staining of the gp96 protein was observed in all glioma tissues and normal brain tissues (Figure 1(a)). However, the extent of staining was highly variable, such that grade IV gliomas exhibited a significantly higher level of gp96 expression than grade II/III gliomas and normal brain tissues (Figure 1(b)). Accordingly, the transcript levels of HSP90B1 (the gene encoding the gp96 protein) were significantly higher in grade IV gliomas than in grade II/III gliomas (Supplemental Figures 1(a) and 1(b)). However, there is no significant difference in gp96 expression among grade II and III gliomas and normal brain tissues (Figure 1(b)). We observed that IDH-wildtype gliomas exhibited higher transcriptional activity than IDH-mutant gliomas regardless of the malignancy grade, but this difference was not observed at the protein level (Supplemental Figures 1(c)–1(h)). Taken together, gp96 was overexpressed in grade IV gliomas, whereas its protein expression was similar among the glioma genetic subtypes. This finding indicates that gp96 is a universe target for the treatment of grade IV gliomas, regardless of their genetic types.

### 3.2. High gp96 Expression Associated with Unfavorable Prognosis in Glioma Patients

**Figure 2:**
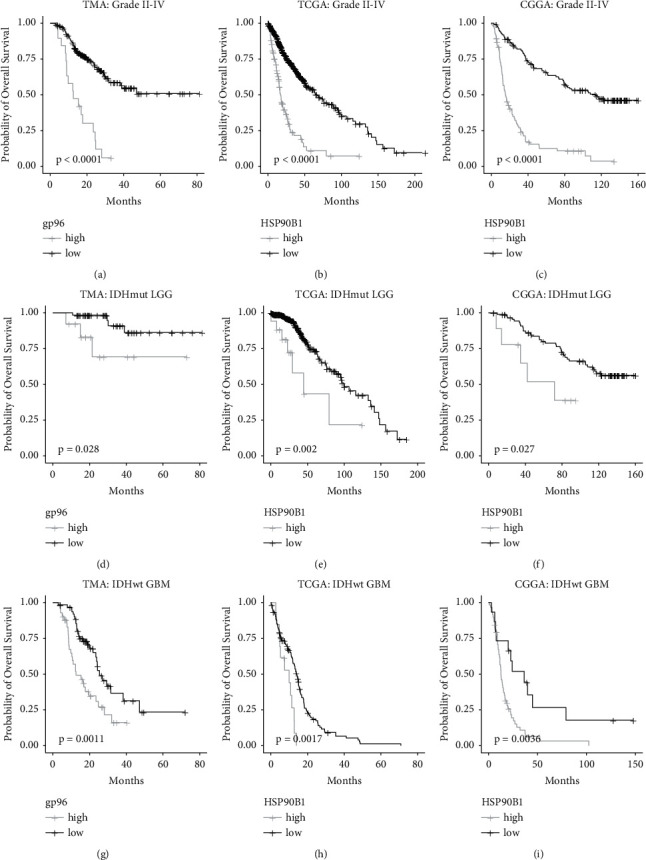
High gp96 expression was associated with unfavorable prognosis. Kaplan–Meier analysis was used to estimate the overall survival (OS), and the log-rank test was applied to estimate between-group OS differences. TMA: gp96 staining extent from the TMA study; TCGA: transcriptional level of HSP90B1 (the gp96 protein-encoding gene) in the TCGA dataset; CGGA: transcriptional level of HSP90B1 in the CGGA dataset; IDHmut LGG : IDH-mutant grade II/III gliomas; IDHwt GBM : IDH-wildtype glioblastomas.

Overexpression of gp96 in grade IV gliomas than grade II/III gliomas suggests that gp96 would be a protein associated with glioma aggressiveness [8]. Therefore, we investigated whether gp96 would be a prognostic marker predicting poor outcomes in glioma patients. Consistent with Hu's study [8], we also observed that higher gp96 expression associated with shorten overall survival time in all malignant gliomas (grade II-IV) (Figure 2(a)), and the TCGA and CGGA analyses verify the association (Figures 2(b) and 2(c)). After controlling the confounding effects by malignancy grade and IDH mutations on patients' survival, we still observed a significant association between high expression of gp96 and unfavorable clinical outcomes (Figures 2(d)–2(i)), reflecting that gp96's high expression has an independent and negative impact on outcomes of glioma patients.

### 3.3. gp96 Expression Positively Correlated with *T* Cell Infiltration Levels

As a master chaperone in immune cells [11, 13, 16], the gp96 protein engages in key immune response activities, such as pathogen defense, immune cell interaction, and immune homeostasis maintenance. However, the influence of gp96 expression on immune infiltrates within the glioma microenvironment remains poorly understood. Therefore, we costained CD8^+^ and CD4^+^ T cells on the TMA with gp96 (Figure 1(a)). Consistent with previous reports [15, 17, 18], GITs were quite sparse in these tissues (Figure 1(a)): the median densities of CD8^+^ and CD4^+^ GITs were only 3.64/mm^2^ and 8.72/mm^2^, respectively. As reported previously [19], the CD4^+^ GIT density was higher than the CD8^+^ GIT density. Furthermore, the GIT densities varied significantly among different glioma grades: grade IV gliomas had a higher GIT density than grade II/III gliomas and normal brain tissues (Figures 1(c) and 1(d)), and IDH-mutant gliomas had a rare immune infiltration as compared with the wildtype (Supplemental Figures 2(a) and 2(b)); both phenomena have already been observed in previous studies [15, 20–23]. All these findings suggest that MIS is an available method for exploring *T* cell infiltration in gliomas.

**Figure 3:**
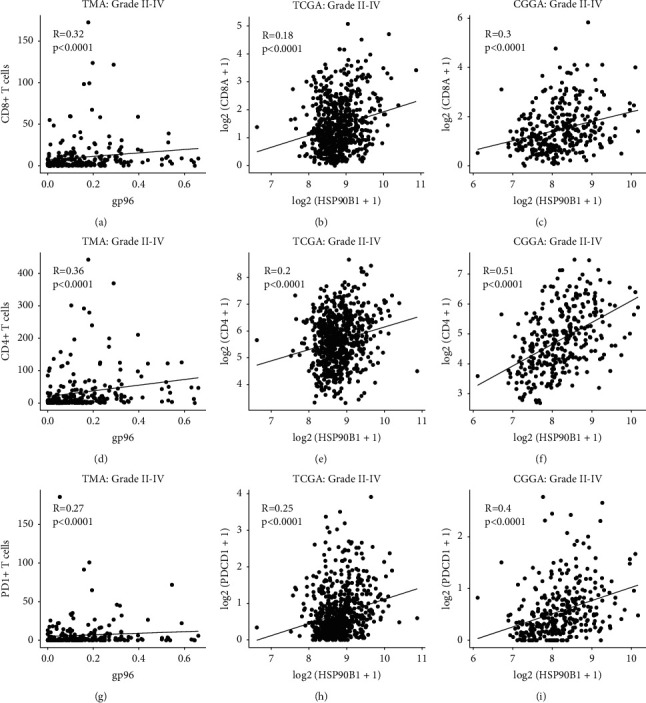
gp96 expression positively correlated with immune cell infiltrative levels. Spearman correlation analysis was utilized to examine the correlations of gp96 expression with CD8^+^ (a–c), CD4^+^ (d–f), and PD-1^+^ (g-i) immune cell infiltration. TMA: results from the TMA; TCGA: results from the TCGA dataset analysis; CGGA: results from the CGGA dataset analysis. CD4, CD8A, and PDCD1 transcriptional levels were used to reflect CD4, CD8, and PD-1 immune cell infiltration, respectively, in the TCGA and CGGA analyses. HSP90B1: the gp96 protein-encoding gene.

Using MIS, we observed a significantly positive correlation of gp96 expression with the infiltration levels of CD8^+^ (Figure 3(a)) and CD4^+^ (Figure 3(d)) GITs in grade II-IV gliomas. Similar results were also obtained upon analyses of datasets from the TCGA and CGGA (Figures 3(b) and 3(c), 3(e) and 3(f)). Since both gp96 expression and GIT densities increased with the malignancy grades (Figures 1(b)–1(d)), we next controlled the confounding effects of malignancy grades for the correlation analysis. As shown in Supplemental Figures (3) and (4), the correlative levels between gp96 expression and GITs were reduced, but still significant in most comparisons after gliomas were further stratified into grade II/III and IV gliomas. This result reflects that these correlations were independent of malignancy grades, suggesting a possible mechanism underlying the positive correlation between gp96 expression and GIT infiltration.

### 3.4. Higher gp96 Expression Associated with Increased Infiltration of CD4^+^ PD-1^+^ GITs

Programmed death-1 (PD-1) expression on GITs usually reflects a state of *T* cell dysfunction [24]. Therefore, we costained PD-1 in the same slide to reveal the dysfunctional status among GITs (Figure 1(a)). Also, grade III or IV gliomas had increased PD-1^+^ immune cell infiltration as compared with grade II gliomas and normal brain tissues (Figure 1(e)). We also observed that tumor gp96 expression correlated significantly with single PD-1^+^ immune cell infiltrative levels within the tumor microenvironment (Figures 3(g)–3(i), Supplemental Figures 3(g)–3(i), Supplemental Figures 4(g)–4(i)), suggesting a possible interplay between gp96 expression and PD-1 upregulation.

**Figure 4:**
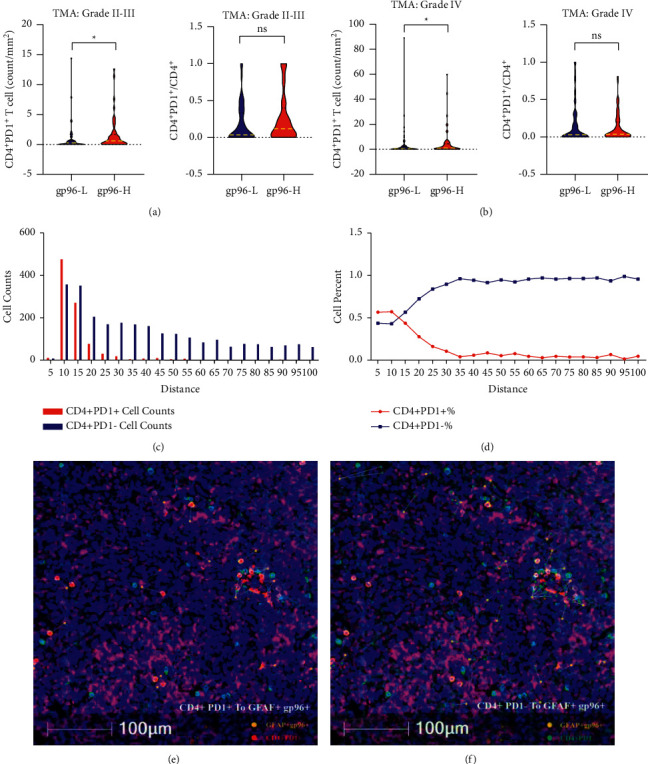
Higher gp96 expression associated with increased infiltration of CD4^+^ PD-1^+^*T* cells. (a, b) CD4^+^ PD-1^+^ T cell densities/proportions in grade II-III (a) and IV (b) gliomas categorized by the extent of gp96 staining (gp96-H: > median value, gp96-L: ≤ median value). (c, d) Counts (c) and proportions (d) of CD4^+^ PD-1^+^ T cells and CD4^+^ PD-1^−^ T cells were calculated for each segment 5 *µ*m away from gp96^+^ GFAP^+^ cells (*n* = 5). (e, f) Representative spatial distribution of CD4^+^ PD-1^+^*T* cells (e) and CD4^+^ PD-1^−^*T* cells (f) within the scope of 100 *µ*m around GFAP^+^ cells (representing glioma cells) with gp96 expression; five GBM specimens with abundant infiltrative CD4^+^*T* cells were utilized for the spatial distribution analysis. TMA: results from the TMA. Mann–Whitney *U* test, *∗p* < 0.05.

Importantly, we observed a higher density of CD4^+^ PD-1^+^ GITs in gp96-high gliomas than in gp96-low gliomas (Figures 4(a) and 4(b)), but this phenomenon did not exist for CD8^+^ PD-1^+^ GITs (Supplemental Figure 5). This finding implies that gp96 expression in tumor is specifically associated with dysfunctional CD4^+^ T cell infiltration. We next utilized a spatial distribution analysis to understand whether gp96 expression in glioma cells would have a locoregional effect on PD-1 expression in CD4^+^ GITs (Figures 4(c)–4(f)). We observed that CD4^+^ PD-1^+^ GITs were accumulated in the regions surrounding gp96^+^ glioma cells, whereas CD4^+^ PD-1^−^ GITs were relatively evenly distributed across the tumor tissues. Therefore, CD4^+^ PD-1^+^ GITs were attracted to region near gp96^+^ glioma cells, further confirming the close association of gp96 expression with dysfunctional CD4^+^ T cell infiltration in gliomas, and suggesting an intrinsic mechanism that requires further investigation.

### 3.5. gp96-Immune Cell Score Classified Glioma Patients into Four Outcome Groups

**Figure 5:**
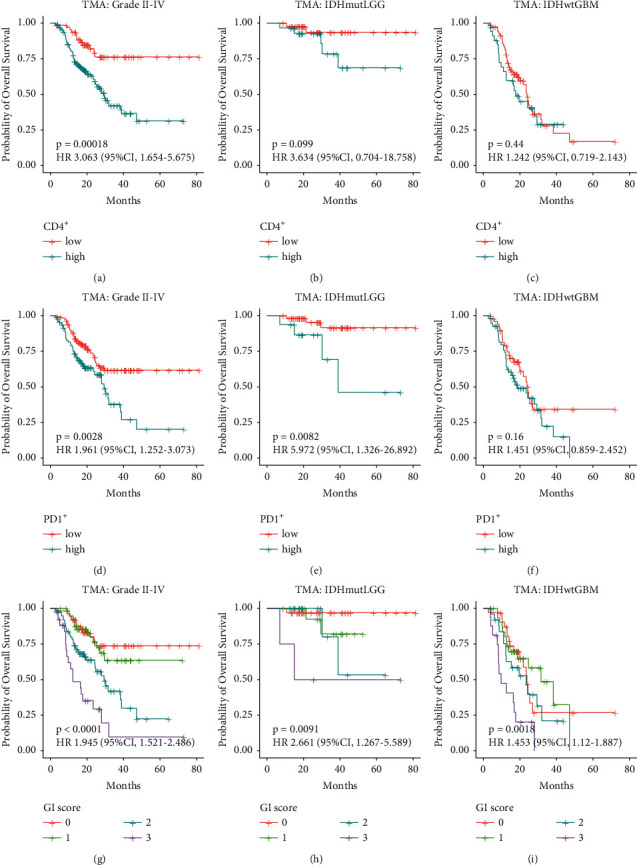
gp96 expression and *T* cell infiltrate levels determined the outcomes of glioma patients. Overall survival (OS) differences among grade II-IV glioma (a, d, g), IDHmut LGG (b, e, h) and IDHwt GBM (c, f, i) patients stratified by CD4+ T cell densities (a–c), PD-1+ immune cell densities (d–f) and the GI score (g–i), respectively. The GI (gp96-immune cell) score was calculated by summing the values that were defined as following: high gp96 expression = 1, low gp96 expression = 0; high CD4+ cell density = 1, low CD4+ cell density = 0; high PD-1+ cell density = 1, low PD-1+ cell density = 0). Kaplan–Meier analysis was used to estimate OS, and the log-rank test was applied to estimate between-group OS differences. IDHmut LGG : IDH-mutant grade II/III gliomas; IDHwt GBM : IDH-wildtype glioblastomas.

Since high expression of gp96 is associated with increased immune cell infiltration within gliomas, we next aimed to investigate the value of combining gp96 expression and immune cell levels in predicting outcomes of glioma patients. We first examined the value of immune cell levels in outcome prediction. Consistent with previous studies [19, 25, 26], increased CD4^+^ and PD-1^+^ cells both predicted worse outcomes in all glioma patients (Figures 5(a) and 5(d)). Furthermore, the prediction by them was still significant for IDH-mutant LGG (lower grade gliomas: grade II/III gliomas, Figures 5(b) and 5(e)), whereas not for IDH-wildtype GBM (Figure 5(c) and 5(f)), reflecting they would be grade-independent prognostic biomarkers for IDH-mutant LGG.

Next, we built an outcome-predictive score, termed the gp96-immune cell score (GI score), by summing the values that were defined as following: high gp96 expression = 1, low gp96 expression = 0; high CD4^+^ cell density = 1, low CD4^+^ cell density = 0; high PD-1^+^ cell density = 1, low PD-1^+^ cell density = 0. With this metric, all malignant glioma patients were stratified into four outcome-distinct groups. The higher GI score represented the worse clinical outcomes: the score 3 group exhibited the poorest outcome, with a median OS of 12.4 months; the score 2 group had a median OS of 29.3 months; whereas the score 1 and 0 groups had relatively favorable outcomes (hazard ratio, 1.945; 95% CI, 1.521–2.486; *P* < 0.0001) (Figure 5(g)).

Furthermore, the prediction for outcomes by GI score was still statistically significant after adjusting for the WHO malignancy grades and IDH mutation status (Figures 5(h) and 5(i)). The GI-score 3 group always represented the worst-outcome gliomas in all comparisons (Figures 5(g)–5(i)). Accordingly, a multivariate Cox analysis further showed that GI-score 3 outperformed the WHO malignancy grade as an independent predictor of unfavorable clinical outcomes (hazard ratio, 3.003; 95% CI, 1.597–5.649; *P* < 0.001) (Supplemental Figure 6).

### 3.6. The Correlation of gp96 Expression with *T* Cell Infiltration Partly Depends on the Interferon Response Pathways

**Figure 6:**
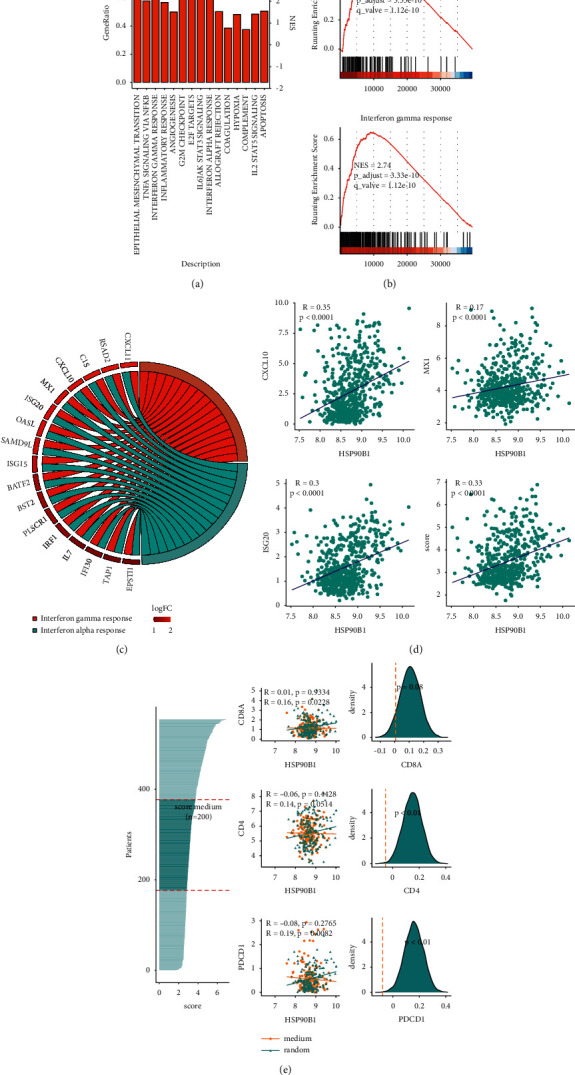
The correlation of gp96 expression with immune infiltrates partly depends on the interferon response pathways. The enrichment of molecular pathways was assessed by GSEA of the DEGs between the 100 samples with the highest gp96 expression and the 100 samples with the lowest gp96 expression in the TCGA datasets. Genes with fold changes > 2 and adjusted *p* values < 0.01 between the two groups were defined as DEGs. (b) IFN-*α* and IFN-*γ* response pathways were highly enriched according to GSEA of the DEGs. (c) Shared DEGs between the IFN-*α* and IFN-*γ* response pathways are shown. (d) Correlations of gp96 expression with CXCL10, MX1, ISG20, and ISG scores were significant. The ISG scores were calculated as the log_2_ (TPM + 0.1) of the shared DEGs to reflect mean activities of the IFN-*α*/*γ* response pathways. (e) Correlations of gp96 expression with CD8A, CD4, and PDCD1 expression were significantly reduced in 200 glioma cases with minimal ISG score variation compared to 200 randomly selected cases.

To explore possible molecular mechanisms associated with gp96 expression, we first obtained DEGs (differentially expressed genes) from the TCGA datasets by comparing the high and low gp96 expression groups (Supplemental Figures 7(a) and 7(b)) and utilized the GSEA (gene set enrichment analysis) to retrieve enriched pathways among the DEGs. Aside from some known pathways supposedly involving gp96, such as the unfolded protein response, epithelial-mesenchymal transition, angiogenesis, and apoptosis [11, 13], the most intriguing finding was that inflammation-related pathways, such as the IFN-*α* response, the IL6-JAK-STAT6 signaling pathway, and the IFN-*γ* response, were highly enriched in gp96-related DEGs (Figures 6(a) and 6(b), Supplemental Figures 7(c) and 7(d)), suggesting an important role of gp96 in regulating the inflammatory response in glioma.

Considering that the IFN-*α* and IFN-*γ* response are two major pathways involved in T cell infiltration, we hypothesized that they would be the underlying mechanisms that mediate the close association between gp96 and GITs. To test this hypothesis, we chose 24 DEGs shared by the IFN-*α* and IFN-*γ* response pathways (Figure 6(c)) and calculated a score, termed the IPA (interferon pathway activity) score, to measure the mean activities of both pathways. As expected, gp96 expression was positively correlated with the IPA score as well as expression of some well-known ISGs, such as CXCL10, MX1, and ISG20 (Figure 6(d)). We then selected a group of 200 gliomas with IPA scores within the medium segment in the TCGA datasets to represent gliomas with comparable IPA scores (IPA score variation was minimized) (Figure 6(e)). Compared to those in the 200 randomly selected gliomas, the correlations of gp96 expression with CD8, CD4, and PD-1 expression were significantly reduced in the IPA-comparable group (Figure 6(e)). Furthermore, this observation was also replicated in the situation when only including grade II-III glioma cases for minimizing confounding from malignancy grade discrepancy in the analysis (Supplemental Figure 7(e)). All these findings indicate that the correlations between gp96 expression and GITs are, at least partly, mediated by the interferon response pathways.

## 4. Discussion

gp96 is the most abundant protein in the ER and is involved in protein folding and assembly, misfolded protein export for degradation, and ER homeostasis maintenance. gp96 overexpression is reportedly linked to increased malignancy in a variety of cancers, since it chaperones key molecules that engage in tumor cell proliferation and invasion, such as insulin-growth factor 1 (IGF1), low-density lipoprotein receptor-related protein 6 (LPR6), and integrins [11, 13]. In glioma, gp96 overexpression has also been reported to be associated with aggressiveness, increased malignancy grades, and worse clinical outcomes [8]. In this study, we confirmed these findings in a much larger cohort of glioma patients. Our results demonstrated that gp96 was overexpressed in grade IV gliomas (Figure 1(b)), and high gp96 expression was associated with unfavorable outcomes, independent of the malignancy grade (Figures 2(d)–2(i)). We also examined differences in gp96 expression among glioma patients grouped by the IDH mutations and/or 1p19q-codeletion status. However, no consistent results were observed between the TMA and TCGA/CGGA analyses, indicating that gp96 expression is quite conserved among these glioma groups. Considering gp96-selective inhibitors and monoclonal antibodies are under development [9–11], our results indicate grade IV gliomas, regardless of its IDH mutation status, are potential indication for this novel therapeutic.

Regarding the underlying mechanisms, the Wnt-*β* catenin pathway is reportedly involved in mediating gp96 expression and promoting glioma aggressiveness [8]. Low-density lipoprotein receptor-related protein 6 (LPR6) is a WNT coreceptor that is supposed to engage in the regulation of the Wnt-*β* catenin pathway by gp96, since it requires assistance from gp96 for its exportation from the ER to the cell surface [10, 26]. However, we also observed that gp96 expression was highly correlated with some well-known protumor pathways, such as epithelial-mesenchymal transition, the G2/M checkpoint and angiogenesis (Figure 6(a)), implying other mechanisms that could be involved in the gp96-mediated promotion of glioma development, thereby warranting further investigation on the exact mechanisms.

As reported in cholangiocellular carcinoma [14] and lung adenocarcinoma [7], we also observed it in gliomas a close association between gp96 expression and *T* cell infiltration. We further revealed that the interferon response pathways are implicated in this association (Figure 6(e)). However, the detailed mechanisms underlying the gp96-mediated activation of the interferon response pathways remain poorly understood. We propose two competing hypotheses to explain the correlation of gp96 expression with interferon response pathway activation. One suggests that gp96 overexpression is only a canonical hallmark of increased ER stress induced by misfolded protein accumulation [13, 16], which also triggers these pathways. Alternatively, gp96 could regulate key molecules of the pathways through its chaperone effect (e.g., assisting in the export of toll-like receptors (TLRs) to the cell surface, thereby upregulating the activities of the pathways). Further studies on glioma cells after gp96 overexpression or knockdown would help test these hypotheses.

Malignant glioma is a heterogenous brain cancer with distinct clinical outcomes, rendering outcome-predictive biomarker discovery necessary for this deadly tumor. We observed both gp96 overexpression and increased CD4/PD-1 immune cell densities associated with shortened OS time in glioma patients (Figures 2 and 5). We then integrated their levels to build the GI score, that was also inversely associated with OS time (Figure 5(g) and Supplemental Figure 6), thus providing a panel of potential biomarkers for the prognostication of glioma patients. Therefore, an expanded glioma cohort is warranted to further validate their predictive accuracies as well as determine the cut-off values.

gp96 can noncovalently bind to tumor antigenic peptides to form a gp96-peptide complex that can be taken up by dendritic cells (DCs) and ultimately trigger an antitumor immune response [27, 28]. Based on this principle, a peptide vaccine, termed the heat shock protein-peptide complex-96 (HSPPC-96) vaccine, was developed to treat a variety of malignancies [29]. The HSPPC-96 vaccine has also shown encouraging results in early clinical studies on recurrent and newly diagnosed GBM [30–33]. Herein, we uncovered the gp96 expression itself as well as its integration with GIT densities, the GI score, were inversely associated with OS time in glioma patients receiving the standard-of-care treatment (Figures 2 and 5). Meanwhile, we also observed that gp96 can promote the infiltration of T cells, especially CD4^+^ PD-1^+^ T cells, partly via the interferon response pathways (Figures 3, 4 and 6). Since gp96 is a key component of the HSPPC-96 vaccine, the impacts of gp96 expression, its association with immune cell infiltration, as well as the underlying mechanisms on the HSPPC-96 vaccine efficacy is an intriguing question that requires further cell experiments as well as clinical studies to answer. Understanding these details will not only provide potential biomarkers that would assist in selecting possibly responsive patients to receive the treatment but would also help us gain deeper insights into vaccine immunity that would facilitate mechanical improvements in this novel immunotherapeutic.

## 5. Conclusion

The overexpression of gp96 was observed in grade IV gliomas, regardless of their IDH mutation status, reflecting gp96 is a universe treatment target for this kind of deadly cancer. gp96 expression and GIT density was highly correlative, that could be mediated by the interferon response pathways. The GI score, that integrates levels of gp96 expression and GIT infiltration, is a potential prognostic classification system for glioma.

## Data Availability

The data of our study were from https://cancergenome.nih.gov/ and www.cgga.org.cn/.

## Supplementary Materials

Supplementary Materials Supplemental Figure 1: gp96 expression and immune cell infiltrative levels in different types of gliomas. (A, B) The gp96 expression among gliomas with WHO malignancy grade II, III, and IV. (C–H). The gp96 expression among three subtypes of grade II/III gliomas: IDH-mutant/1p19q-codeletion (mIDH+1p19q(+)), IDH-mutant/non-1p19q-codeletion (mIDH+1p19q(-)), and IDH-wildtype (wtIDH), and between two subtypes of grade IV gliomas: IDH-mutant (mIDH) and IDH-wild-type (wtIDH). TMA: gp96 protein staining from the TMA; TCGA: transcriptional level of HSP90B1 (the gp96 protein-encoding gene) in the TCGA dataset; CGGA: transcriptional level of HSP90B1 in the CGGA datasets. Mann–Whitney *U* test for two-group comparisons; Kruskal–Wallis test, and Bonferroni post hoc method for multiple comparisons; ^*∗∗*^*p* < 0.01, ^*∗∗∗*^*p* < 0.001. Supplemental Figure 2: T cell infiltration level was higher in IDH-wildtype gliomas than in IDH-mutant gliomas. IDH-wildtype (wtIDH) gliomas exhibit increased CD8^+^ (A) and CD4^+^ (B) T cell infiltration relative to IDH-mutant (mIDH) gliomas. Mann–Whitney *U* test. Supplemental Figure 3: correlation of gp96 expression with immune cell infiltrative levels in grade II-III gliomas. Spearman correlation analysis was utilized to examine the correlations of gp96 expression with CD8+ (A–C), CD4+ (D–F), and PD-1+ (G–I) immune cell infiltration. TMA: results from the TMA; TCGA: results from the TCGA dataset analysis; CGGA: results from the CGGA dataset analysis. CD4, CD8A, and PDCD1 transcriptional levels were used to reflect CD4, CD8, and PD-1 immune cell infiltration, respectively, in the TCGA and CGGA analyses. HSP90B1: the gp96 protein-encoding gene. Supplemental Figure 4: correlation of gp96 expression with immune cell infiltrative levels in grade IV gliomas. Spearman correlation analysis was utilized to examine the correlations of gp96 expression with CD8^+^ (A–C), CD4^+^ (D–F), and PD-1^+^ (G–I) immune cell infiltration. TMA: results from the TMA; TCGA: results from the TCGA dataset analysis; CGGA: results from the CGGA dataset analysis. CD4, CD8A, and PDCD1 transcriptional levels were used to reflect CD4, CD8, and PD-1 immune cell infiltration, respectively, in the TCGA and CGGA analyses. HSP90B1: the gp96 protein-encoding gene. Supplemental Figure 5: difference of CD8^+^ PD-1^+^ T cell infiltration between the high and low gp96 expression gliomas. CD8^+^ PD-1^+^ T cell densities/proportions in grade II-III (A) and IV (B) gliomas categorized by the extent of gp96 staining (gp96-H: > median value, gp96-L: ≤ median value). Supplemental Figure 6: a multivariate Cox regression model predicting median overall survival in glioma patients. Supplemental Figure 7: IFN-*γ*/*α* response pathways were enriched in differentially expressed genes between the high and low HSP90B1 expression groups of gliomas in the TCGA datasets. (A) Glioma samples with the top 100 and bottom 100 transcriptional levels of HSP90B1 (the gp96 protein-encoding gene) were selected and grouped into the high and low HSP90B1 expression groups. (B) Genes with fold-changes > 2 and adjusted *p* values < 0.01 between the two groups were defined as DEGs. (C) IFN-*γ* and IFN-*α* response pathways were significantly enriched in DEGs in the LGG portion of the TCGA datasets. (D) The IFN-*γ* response pathway was significantly enriched in DEGs in the GBM portion of the TCGA datasets. LGG: lower grade (grade II-III) glioma. (E) Correlations of gp96 expression with CD8A, CD4, and PDCD1 expression were significantly reduced in 150 grade II-III glioma cases with minimal ISG score variation compared to 150 randomly selected cases.
