# Supplementary material for: gp96 Expression in Gliomas and Its Association with Tumor Malignancy and T Cell Infiltrating Level
Source: J Oncol. 2022 Jun 26;2022:9575867. doi: 10.1155/2022/9575867 (PMC9251151; doi:10.1155/2022/9575867)
Supplement: Supplementary Materials — Supplemental Figure 1: gp96 expression and immune cell infiltrative levels in different types of gliomas. (A, B) The gp96 expression among gliomas with WHO malignancy grade II, III, and IV. (C–H). The gp96 expression among three subtypes of grade II/III gliomas: IDH-mutant/1p19q-codeletion (mIDH+1p19q(+)), IDH-mutant/non-1p19q-codeletion (mIDH+1p19q(-)), and IDH-wildtype (wtIDH), and between two subtypes of grade IV gliomas: IDH-mutant (mIDH) and IDH-wild-type (wtIDH). TMA: gp96 protein staining from the TMA; TCGA: transcriptional level of HSP90B1 (the gp96 protein-encoding gene) in the TCGA dataset; CGGA: transcriptional level of HSP90B1 in the CGGA datasets. Mann–Whitney U test for two-group comparisons; Kruskal–Wallis test, and Bonferroni post hoc method for multiple comparisons; ∗∗p < 0.01, ∗∗∗p < 0.001. Supplemental Figure 2: T cell infiltration level was higher in IDH-wildtype gliomas than in IDH-mutant gliomas. IDH-wildtype (wtIDH) gliomas exhibit increased CD8+ (A) and CD4+ (B) T cell infiltration relative to IDH-mutant (mIDH) gliomas. Mann–Whitney U test. Supplemental Figure 3: correlation of gp96 expression with immune cell infiltrative levels in grade II-III gliomas. Spearman correlation analysis was utilized to examine the correlations of gp96 expression with CD8+ (A–C), CD4+ (D–F), and PD-1+ (G–I) immune cell infiltration. TMA: results from the TMA; TCGA: results from the TCGA dataset analysis; CGGA: results from the CGGA dataset analysis. CD4, CD8A, and PDCD1 transcriptional levels were used to reflect CD4, CD8, and PD-1 immune cell infiltration, respectively, in the TCGA and CGGA analyses. HSP90B1: the gp96 protein-encoding gene. Supplemental Figure 4: correlation of gp96 expression with immune cell infiltrative levels in grade IV gliomas. Spearman correlation analysis was utilized to examine the correlations of gp96 expression with CD8+ (A–C), CD4+ (D–F), and PD-1+ (G–I) immune cell infiltration. TMA: results from the TMA; TCGA: results from the TCGA [file 9575867.f1.zip › 9575867.f1/Supplemental table 1.docx]

| **Supplement Table 1A: Primary antibody used in Multiplex staining** | | | | | |
| --- | --- | --- | --- | --- | --- |
| **Stain Order** | **Antibody** | **Clone or lot# (host)/Company** | **Dilution** | **Opal dye (Dilution)** | **CITY/Nation** |
| **1** | CD8 | ab4055(Rabbit)/Abcam | 1/400 | 540 (1:100) | Cambridge/UK |
| **2** | PD-1 | NAT105 (Mouse)/Abcam | 1/400 | 650 (1:100) | Cambridge/UK |
| **3** | gp96 | ab3674 (Rabbit)/Abcam | 1/400 | 570 (1:100) | Cambridge/UK |
| **4** | GFAP | EPR1034Y (Rabbit)/Abcam | 1/400 | 690 (1:100) | Cambridge/UK |
| **5** | CD4 | EPR6855 (Rabbit)/Abcam | 1/400 | 620 (1:100) | Cambridge/UK |
| **6** | MX1 | ab95926 (Rabbit)/Abcam | 1/200 | 520 (1:100) | Cambridge/UK |
| **7** | DAPI | C0060/Solarbio | 1/500 |  | Beijing/China |

| **Supplement Table 1B: Experimental conditions and procedures of Multiplex staining** | | | | | | | |
| --- | --- | --- | --- | --- | --- | --- | --- |
| **Antigen** | **CD8** | **PD-1** | **gp96** | **GFAP** | **CD4** | **MX-1** | **DAPI** |
| **Antigen retrieval** | AR6  20 min | AR6  20 min | AR9  20 min | AR6  20 min | AR9  20 min | AR6  20 min |  |
| **Wash** | TBST  10 min | TBST  10 min | TBST  10 min | TBST  10 min | TBST  10 min | TBST  10 min |  |
| **Blocking** | 10 min  @RT | 10 min  @RT | 10 min  @RT | 10 min  @RT | 10 min  @RT | 10 min  @RT |  |
| **Inc. with Primary antibody** | 60 min  @37℃ | Over night  @4℃ | Over night  @4℃ | 60 min  @37℃ | Over night  @4℃ | Over night  @4℃ | 10 min  @RT |
| **Wash** | TBST  10 min | TBST  10 min | TBST  10 min | TBST  10 min | TBST  10 min | TBST  10 min | TBST  10 min |
| **Inc. with Opal HRP Polymer Ms+Rb** | 10 min  @RT | 10 min  @RT | 10 min  @RT | 10 min  @RT | 10 min  @RT | 10 min  @RT |  |
| **Wash** | TBST  10 min | TBST  10 min | TBST  10 min | TBST  10 min | TBST  10 min | TBST  10 min | Mounting |
| **Inc. with Opal Dye** | opal 540  10 min  @RT | opal 650  10 min  @RT | opal 570  10 min  @RT | opal 690  10 min  @RT | opal 620  10 min  @RT | opal 520  10 min  @RT |  |
| **Wash** | TBST  10 min | TBST  10 min | TBST  10 min | TBST  10 min | TBST  10 min | TBST  10 min |  |
| Abbreviations: AR6: Antigen retrieval at pH=6; AR9: Antigen retrieval at pH=9; @: at; RT: at Room temperature; Inc: Incubation; Temp: Temperature; Ms+Rb: Mouse and Rabbit | | | | | | | |
